# Neural cell engraftment therapy for sporadic Creutzfeldt-Jakob disease restores neuroelectrophysiological parameters in a cerebral organoid model

**DOI:** 10.1186/s13287-023-03591-2

**Published:** 2023-12-05

**Authors:** Katie Williams, Simote T. Foliaki, Brent Race, Anna Smith, Tina Thomas, Bradley R. Groveman, Cathryn L. Haigh

**Affiliations:** 1 Laboratory of Neurological Infections and Immunity, National Institute of Allergy and Infectious Diseases, Division of Intramural Research, Rocky Mountain Laboratories, National Institutes of Health, 903 South 4Th Street, Hamilton, MT 59840 USA; 2 Rocky Mountain Veterinary Branch, National Institute of Allergy and Infectious Diseases, Division of Intramural Research, Rocky Mountain Laboratories, National Institutes of Health, 903 South 4Th Street, Hamilton, MT 59840 USA

**Keywords:** Prion, Sporadic CJD, Cerebral organoid, Neural progenitor, Electrophysiology, Cell therapy

## Abstract

**Background:**

Sporadic Creutzfeldt-Jakob disease (sCJD), the most common human prion disease, is a fatal neurodegenerative disease with currently no treatment options. Stem cell therapy for neurodegenerative diseases is emerging as a possible treatment option. However, while there are a few clinical trials for other neurodegenerative disorders such as Parkinson’s disease, prion disease cell therapy research has so far been confined to animal models.

**Methods:**

Here, we use a novel approach to study cell therapies in sCJD using a human cerebral organoid model. Cerebral organoids can be infected with sCJD prions allowing us to assess how neural precursor cell (NPC) therapy impacts the progression of sCJD. After 90 days of sCJD or mock infection, organoids were either seeded with NPCs or left unseeded and monitored for cellular composition changes, prion infection parameters and neuroelectrophysiological function at 180 days post-infection.

**Results:**

Our results showed NPCs integrated into organoids leading to an increase in neuronal markers and changes in cell signaling irrespective of sCJD infection. Although a small, but significant, decrease in protease-resistant PrP deposition was observed in the CJD-infected organoids that received the NPCs, other disease-associated parameters showed minimal changes. However, the NPCs had a beneficial impact on organoid function following infection. sCJD infection caused reduction in neuronal spike rate and mean burst spike rate, indicative of reduced action potentials. NPC seeding restored these electrophysiological parameters to the uninfected control level.

**Conclusions:**

Together with the previous animal studies, our results support that cell therapy may have some functional benefit for the treatment of human prion diseases.

**Supplementary Information:**

The online version contains supplementary material available at 10.1186/s13287-023-03591-2.

## Introduction

Human prion diseases are progressive neurodegenerative disorders of which sporadic Creutzfeldt-Jakob disease (sCJD) is the most common manifestation. Prion diseases have varied clinical courses but are inevitably fatal. Currently, there are no approved therapeutic compounds or strategies that show efficacy against disease progression and patient treatments focus on management of symptoms. The causative agents of prion diseases are mis-folded conformers (prions) of a normal cellular protein called the prion protein (PrP). Once mis-folded, prions are able to recruit and mis-fold more PrP in an ongoing cycle until the brain is deleteriously overwhelmed. Most therapeutic options investigated to date have focused on halting or slowing the cycle of mis-folding; however restoring cognitive function and therefore quality of life for the patient is equally important.

Various anti-prion therapeutics have been trialed in humans. Pentosan polysulfate (PPS) and doxycycline have been reported to extend survival time in some patient studies [1–4], but were found ineffective in others [1, 5, 6]. No beneficial changes in cognition or improvement of symptoms were reported in these studies. Quinacrine showed no survival benefit for patients [7–10] but a couple of studies found small improvements in cognitive assessments [8, 11]. Flupirtine was reported to reduce cognitive decline, with improvements in memory and orientation but not speaking tasks (as determined by the ADAS-Cog test, which includes parameters such as word recall, word recognition and spoken language tasks) compared with a placebo group. Despite this there was no survival benefit from the treatment [12]. Overall, anti-prion treatments have at best produced minor improvements in cognition. Since most treatments will be started after the onset of symptoms, identification of therapeutic avenues that can recover cognitive function from a more advanced stage of disease and therefore provide patients with improved quality of life is needed.

One potential avenue for recovering cognitive function is the use of neural progenitor cells (NPCs). This has been trialed in a single Parkinson’s patient who, at the time of publication, had stabilized or improved clinical measurements within 18–24 months of the transplant [13]. A number of clinical trials are currently recruiting participants for, or are actively investigating the effectiveness of, this treatment avenue in Parkinson’s disease. There are also indications from the literature that progenitor cell transplantation could be beneficial in prion diseases. For example, fetal cell grafts delivered by intracerebral (IC) injection into the midline of the frontal cortex improved neuronal retention in late-stage scrapie prion infected mice [14]. Similarly, IC injection of human mesenchymal stem cells into the hippocampus or thalamus [15, 16] and fetal neural stem cells into the hippocampus [17], or intravenous infusion of mesenchymal stem cells [15], were shown to extend the survival time of mice infected with scrapie. A study delivering mesenchymal stromal cells intranasally to mice infected with prions also found that, despite no change in disease incubation period, treated mice showed initially less spongiform change and decreased neuroinflammation throughout the brain [18]. Furthermore, in a mouse model of E200K genetic CJD, transplantation of progenitors significantly delayed disease onset and progression in both initial and advanced clinical states [19, 20]. Despite these promising animal studies, NPC therapy has never been investigated in the context of human prion infection before, likely due to the limited availability of a manipulable human model in which to prove the concept.

We have previously shown that human cerebral organoids (COs) can be infected with sCJD prions and faithfully propagate the original inoculum [21, 22]. The infection could be reduced by treatment with PPS either administered before and during infection or after establishment of infection, supporting the use of the organoid model for screening putative therapeutic strategies [23]. An advantage to the organoid model is that neuronal function during infection and treatment can be measured using neuroelectrophysiology [24, 25]. In this way, it can be determined if a treatment has the potential to improve neuronal function. Induced pluripotent stem cells (iPSC) can be differentiated into multiple cell types including COs and NPCs. We hypothesized that supplementing sCJD-infected COs with NPCs may help reduce the loss of neuroelectrophysiological function, providing evidence that therapeutic avenues to restore function might be worth pursuing in humans.

## Methods

### Human-induced pluripotent stem cells and culture

KYOU-DXR0109B (ACS-1023; ATCC) and IPSC1030 (iPSC β-ACTIN GFP; Sigma-Aldrich) hu-iPSCs were routinely cultured on low growth factor Matrigel (Roche) in mTeSR Plus medium (Stem Cell Technologies) with 5% CO_2_ at 37 °C in a humidified incubator as described in the mTeSR handbook. Colonies were passaged at approximately 70–80% confluency before colonies had started to contact each other.

### Human cerebral organoid generation and routine culture

COs were generated using Stem Cell Technologies cerebral organoid kit. Briefly, for embryoid body (EB) production half of a 70–80% confluent 25 cm^2^ flask of hu-iPSCs was used to seed a 96-well plate. Cells were plated in 50 µl EB media and additionally supplemented on day 0 with 50 μM Y27632. EBs were fed an additional 50 μl of EB media on days 2 and 4. EBs were transferred into neural induction medium (1× glutamax, 1× non-essential amino acids, 1% [v/v] N2, and 1 μg/ml heparin in DME-F12 medium) in low adhesion plates on day 5. Organoids were embedded in Matrigel (Corning) 2 days after neural induction when neuroectoderm was visible. Matrigel embedding was performed using organoid embedding sheets with 20 μl of Matrigel and allowed to set for 30 min at 37 °C. Organoids were then washed into a 6 well low attachment plate and incubated in cerebral organoid maintenance media (1× glutamax, 1× penicillin–streptomycin solution, 0.5× non-essential amino acids, 0.5% [v/v] N2, 1 μl/4 ml insulin, and 1 μl/286 ml 2-Merceptoethanol in 1:1 Neurobasal:DME-F12 medium) with 1% (v/v) B27 minus retinoic acid. On day 10, organoids were transferred into cerebral organoid maintenance medium with 1% (v/v) B27 plus retinoic acid for long-term culture. Long-term agitated culture was performed in 125-ml Erlenmeyer flasks on an orbital shaker at 85 rpm. Media was changed 1–2 times weekly.

### NPC differentiation and NPC transplanted to COs

Neuronal Progenitor Cells were generated using Neural Induction Kit with SMAD inhibitors (Stem Cell Technologies). Briefly, iPSC cells expressing GFP were plated at 1.3 × 10^6^ with neural induction media plus SMADi. Cells were grown and passaged 3 times in neural induction media with SMADi. After the third passage, cells were frozen in neural induction media with SMADi and 10% DMSO until experiments were started. 11 days prior to embedding COs NPCs were thawed and passaged one additional time in neural induction media with SMADi.

NPCs were collected using accutase and centrifuged at 300 × g for 5 min. Cells were then counted using the Muse cell count and viability reagent a Muse flow cytometer (Guava Instruments), which showed viability to be ~ 92%. Cells were mixed with Matrigel to provide 100,000 viable cells per 20 µl with a 1:2 ratio of cell media to Matrigel. Organoids were transferred to organoid embedding sheets and extra media was removed. Twenty µl of cell/ Matrigel mixture was then pipetted onto to each organoid. Organoids were rolled in Matrigel using a pipette tip to coat and incubated at 37 °C for 20–30 min to allow the Matrigel to solidify after which time organoids were then transferred back into flasks in organoid maintenance media with vitamin A. Organoids not receiving NPC treatment were embedded in a Matrigel/media mix void of cells using the same protocol described above.

### Prion infections of human COs

Brain homogenates from sporadic CJD subtype MV2 or a normal brain homogenate (NBH) were diluted into organoid maintenance media to a final concentration of 0.1% (tissue wet weight/volume). At the start of infection, existing media was removed from the organoids and replaced with the inoculated media. Twenty-four hours after inoculation, an equivalent volume of fresh media was added to the cultures (diluting the original inoculum 1 in 2). A full media and culture vessel exchange was performed 7 days after initial exposure. Organoids were maintained in agitated culture with weekly media changes. All brain tissues used in this study were obtained on autopsy and were therefore exempt from review by the NIH Office of Human Subjects Research Protection.

### Prestoblue analysis and LDH

Prestoblue metabolism was measured as per the manufacturer’s instructions from 4 to 6 random representative organoids from each group prior to infection, prior to NPC treatment, 2 weeks following treatment and at the conclusion of the study. Briefly, prestoblue reagent was diluted 1 in 10 in organoid media. Existing organoid media was removed and organoids were incubated in prestoblue-containing media for 30 min. The metabolized prestoblue-containing media was then transferred into replicate wells for analysis. Prestoblue fluorescence was measured at 560 nm excitation and 590 nm emission in a ClarioStar plate reader (BMG). Lactate dehydrogenase (LDH) was measured from 12 organoids per group at the ~ 180 dpi timepoint. Twenty-four hours prior to LDH measurement, organoids were separated into 24 well plates with 0.5 ml of fresh media per organoid/per well. One hundred µl of this 24-h old media was then mixed with 100 µl of LDH, dye and catalyst, incubated at 37 °C for 15 min and developed with 50 µl LDH stop solution, before absorbance was measured on the ClarioStar plate reader (BMG) at 460 nm (with reference wavelength 690 nm subtracted from the reading).

### RT-QuIC

Real-time QuIC (RT-QuIC) assays were performed similarly to those reported previously [21]. Briefly, the RT-QuIC reaction mix contained 10 mM phosphate buffer (pH 7.4), 300 mM NaCl, 0.1 mg/ml hamster recombinant PrP 90–231, 10 μM thioflavin T (ThT), 0.002% SDS (from the homogenate dilution), and 1 mM ethylenediaminetetraacetic acid tetrasodium salt (EDTA). Organoids were homogenized by motorized pestle to 10% (w/v) in PBS and cleared with a 2000 × g 2 min centrifugation. Organoid homogenates were serially diluted in 0.1% SDS/PBS/N2 solution, and 1 μl was loaded into each well of a black 384-well plate with a clear bottom (Nunc) containing 49 μl of reaction mixture. Plates were sealed (Nalgene Nunc International sealer) and incubated in a BMG FLUOstar Omega plate reader at 50 °C for 50 h with cycles of 60 s of shaking (700 rpm, double-orbital) and 60 s of rest throughout the incubation. ThT fluorescence measurements (excitation, 450 ± 10 nm; emission, 480 ± 10 nm [bottom read]) were taken every 45 min. Spearman–Kärber analyses was used to provide estimates of the concentrations of seeding activity units giving positive reactions in 50% of replicate reactions, i.e., the 50% “seeding doses” or SD_50_’s as previously described [21].

### Proteinase-K digests and western blotting

10% organoid homogenates were treated with 10 μg/ml Proteinase-K in 1% Sarkosyl for 1 h at 37 °C with 400 rpm shaking. The reactions were stopped by incubation with 1 μM Pefabloc for 5 min at 4 °C. Samples were then mixed with 2 × Bolt LDS sample buffer (Invitrogen) containing 6% β-mercaptoethanol and boiled for 5 min. For western blots that were not PK treated, samples were mixed with sample buffer and boiled for 5 min. Samples were run on Bolt 4–12% Bis–Tris gels (Invitrogen) and transferred to PVDF membranes using the iBlot 2 transfer system (Invitrogen). Antibodies were used at the following concentrations, PSD-95 1:5000, doublecortin (Abcam) 1:2000, sox2 (Cell Signaling Technologies) 1:5000, oxidative stress cocktail 1:5000, GFAP (Abcam) 1:2000, 3F4 1:5000 (Millipore), AKT (Cell Signaling Technologies) 1:1000. Secondary antibodies form Abcam were goat anti-mouse 1:5000 and goat anti-rabbit 1:5000. Bands were visualized using SuperSignal West Atto Ultimate Sensitivity Chemiluminescent Substrate (Invitrogen) and imaged on the iBright imaging system (Invitrogen). Blots were normalized as a ratio of target to total protein using Coomassie stain, and quantification was preformed using Image J.

### Multi-electrode arrays (MEA) recording

COs were adhered to the MEA using 0.005–0.01% polyethyleneimine (PEI) and 2 μg/mL laminin as described previously (PMID: 32,976,764). Each well of 24-multi-well MEA (multi-channel systems) was pre-coated with 100 µL PEI for 1 h at room temperature, washed three times with MilliQ water (5 min/wash), and allowed to air dry. Once dried, the wells were coated with Laminin by adding 20 µL of 2 µg/mL Laminin (Corning) to each well to cover all electrodes and incubating in Laminin for ~ 1 h at room temperature. The wells were washed three times with MilliQ water and filled with ~ 800 µL BrainPhys media (Stem Cell Technologies). The COs were then dropped into pre-coated wells (about one organoid/well) and gently moved to the center of the well to ensure good contact with the electrodes. Plated COs were incubated without shaking in a 5% CO_2_ incubator for at least 18 h before reading the neuronal network activity by a multiwell MEA system (multi-channel systems). Local field potential was recorded at a sampling frequency of 20 kHz and filtered using a second-order Butterworth high-pass filter (300 Hz) and a fourth-order Butterworth low-pass filter (3500 Hz). Spikes were detected as local field potential peaks (positive or negative) greater than 3.6 standard deviations of the mean local field potential noise level. A cluster of spikes from a single electrode was considered a burst when at least four spikes were detected within 100 ms. A network burst was detected when at least three electrodes showed overlapping bursts. An electrode detecting less than 250 spikes per minute was considered inactive.

### Immunohistochemistry and immunofluorescence

Five COs from each experimental group were submitted for histologic studies. Organoids were immersed in 3.7% neutral buffered formalin for ~ 24 h prior to standard embedding in paraffin. Five-micron sections were cut, stained and examined by routine H&E staining for overall pathology. IHC staining specifically for prion protein was performed using anti-PrP antibody SAF32 (Cayman Chemical) [26]. De-paraffinization, antigen retrieval and staining were performed using the Discovery Ultra-Staining Module. Antigen retrieval for SAF32 staining was achieved using extended cell conditioning with CC1 buffer (Ventana) containing Tris–Borate-EDTA, pH 8.0 for 64 min at 95 °C. Prior to staining, a horse serum blocker (Vector #136,021) was applied at 37°C for 20 min. To stain PrP, we applied SAF32 at a dilution of 1:2,000 in antibody dilution buffer (Ventana) for 1 h at 37 °C. The secondary antibody, horse anti-mouse IgG (Vector#30,129) was applied undiluted for 32 min at 37 °C. Detection was performed with ChromoMap DAB (Roche/Ventana #NC1859896). All histopathology slides were analyzed and photographed Aperio Imagescope software.

NPCs for characterization were grown overnight in a chamber slide before being fixed with 3.7% neutral buffered formalin for 15 min. Chamber slides were then rinsed with PBS before staining with Sox2 (Cell Signaling Technologies) 1:1000, Doublecortin (Abcam) 1:2000, NFL (Santa Cruz Biotechnology) 1:100, and Dapi 1:10,000. NPC characterization cells were then counted and expressed as a ratio of positive target cells to Dapi from the same field.

Organoid Map2 staining was performed on de-paraffined, antigen retrieval sections using the same protocol as above. Map2 (Synaptic Systems) was used at 1:200. GFP visualization of NPCs were done on live organoids. All fluorescent imaging was performed with an EVOS microscope using the same exposure settings between groups and quantification was done using ImageJ. Map2 quantification was from one 4× image per organoid with five organoids represented per group and was normalized to size of organoid.

General CO characterization was performed on 6- to 11-month-old organoids. Immunohistochemistry fixation, embedding and sectioning were performed as described above. IHC staining for neuronal and astrocyte markers were preformed using the following dilutions: Sox2 (Cell Signaling Technologies) 1:200, doublecortin (Abcam) 1:5000, NFL (Santa Cruz Biotechnology) 1:500, Map2 (Abcam) 1:2500, GFAP (Dako) 1:3500. Appropriate secondaries for IHC where undiluted either horse anti-mouse or horse anti-rabbit (Vector). Immunofluorescence were performed on frozen cryosections and imaged using a confocal. Primary antibodies, NFL, B Tubulin, and F-actin were used at 1:200 and DAPI was 1:10,000. Appropriate secondary antibodies were used at 1:500.

### Bioplex

Organoids were triturated in 200 µl cell lysis buffer including PMSF and factor QG (BioRad). Samples were then frozen until later analysis were performed. Once thawed samples were cleared of debris by centrifuging at 15,000 × g for 10 min at 4 °C. Cleared samples were then analyzed using a BCA assay and further diluted in cell lysis buffer to ensure all samples were in a range appropriate for the BioPlex cell signaling assays, around 200 µg/ml. BioPlex cell signaling panels MAPK 9-plex and AKT 8-plex panels were run according to BioPlex instructions on the BioPlex 3D suspension array system. Mean fluorescents for each analyte were normalized to total protein as determined by an average from 2–3 independent BCA runs.

## Results

Ideally, treatment regimens for neurodegenerative diseases should include restoration of neuronal function. We previously showed that COs can be used to examine compounds that inhibit prion replication using the anti-prion compound PPS [23]. Unfortunately, PPS treatment, while having a stimulatory effect on neuroelectrophysiology in uninfected COs, does not recover function of prion infected organoids (Additional file 1: Fig. S1). NPC transplantation has previously been shown to extend survival time in murine prion diseases [14–17], and as NPCs have the ability to integrate into the tissue and form new neurons [27], we hypothesized that restoration of function might be possible following NPC augmentation of organoids in culture.

### NPCs can be seeded into COs

**Fig. 1:**
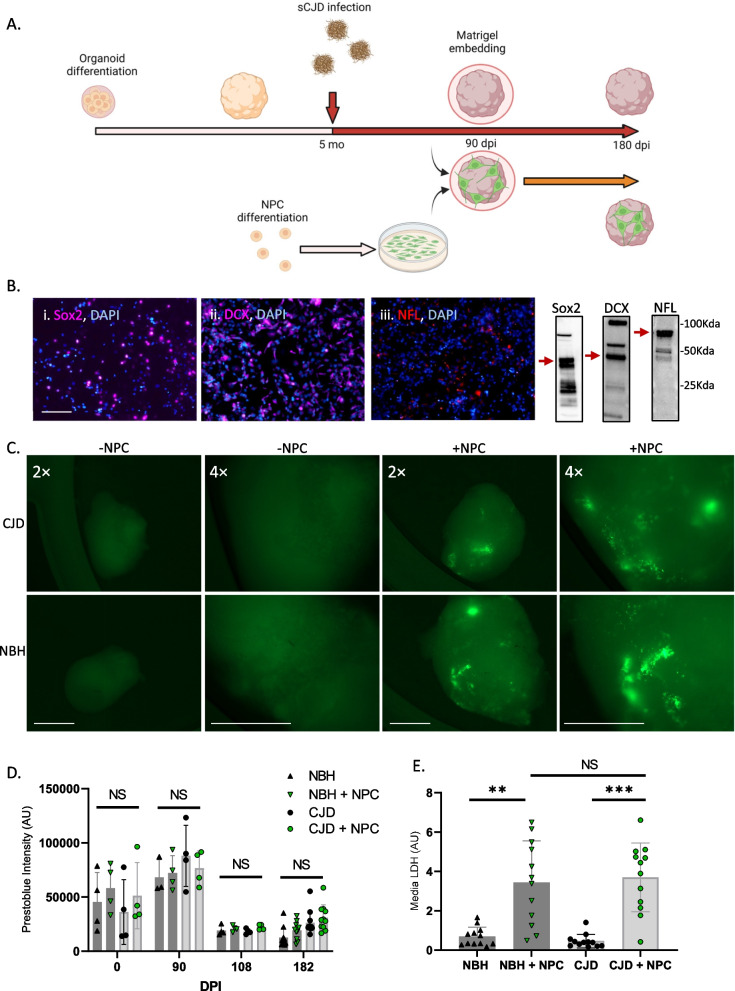
Input NPC characterization and organoid health. **A** Schematic of the experimental approach (created with Biorender). **B** Immunofluorescent staining (left) and western blot (right, uncropped blots are shown in Additional file 1: Fig. S3) of input NPCs with Sox2 (i.), DCX (ii.), and NFL (iii.). Scale bar = 100 µm. **C** GFP fluorescence of live organoids with and without NPCs at 184 dpi (94 days post-seeding). Scale bar = 1000 µm for both magnifications. **D **Prestoblue viability assay and **E** media LDH analysis of representative organoids. Prestoblue measurements were taken at various time points throughout infection (*n* = 4 at 0–108 dpi, n = 12 at 182dpi), and LDH measurements are 182dpi (*n* = 12). Each dot represents an individual organoid with shaded bars showing the mean and error bars showing the SD. Statistics were performed at each dpi using Kruskal–Wallis test with Dunn’s correction for multiple comparisons. **p < 0.01, ***p < 0.001, NS = Not significant

To determine if NPCs might be able to rescue certain disease parameters, we devised an approach to introduce NPCs after infection of the organoids with sCJD prions at a point where significant prion propagation had already occurred. Human COs were differentiated from iPSCs using the Lancaster and Knoblich protocol [28] that produces organoids with populations of mature neurons, astrocytes and oligodendrocytes, with depletion of the progenitor populations over time (characterized in [24, 25, 29] and Additional file 1: Fig. S2). COs were grown to 5 months old and then either infected with CJD or exposed to a mock infection with normal brain homogenate (NBH). At 90 days post-infection (dpi), organoids were seeded with NPCs or left unseeded for the remainder of the 184-day incubation period (Fig. 1A). NPCs were differentiated from iPSCs engineered to constitutively express GFP, allowing their integration into the organoids to be monitored. Immunofluorescence and western blot characterization of the NPCs at the time of embedding (Fig. 1B, uncropped blots are shown in Additional file 1: Fig. S3) showed that the majority expressed either Sox2 (a marker of undifferentiated stem cells) or doublecortin (DCX, an early neuronal marker), with lesser populations expressing neurofilaments (NFL that shows progressed differentiation into more mature neurons). Thus, the population is a mixture of multipotent progenitors and neuronally committed cells that for simplicity will continue to be referred to collectively as NPCs. Following integration into the COs, GFP expressing cells can be seen mostly around the periphery of the organoids, as the autofluorescence of the organoid core is too intense to determine the presence of deeper cells and can be observed throughout the experiment until harvest at 184 dpi (Fig. 1C). Organoids were monitored throughout the experiment for cell viability using the prestoblue cell viability assay, which measures metabolism, and at the experimental endpoint using media LDH levels (Fig. 1D and E). The media LDH did indicate a variable increase in cell death in the NPC-seeded COs, suggesting that some of the seeded cells may not survive long term; however, this was not significantly different between the NBH and CJD-infected COs and was not seen in the prestoblue viability assay. Thus, despite the increased media LDH indicating some increase in cell death, the seeded cells persisted and the organoids remained viable for the duration of the incubation period.

**Fig. 2:**
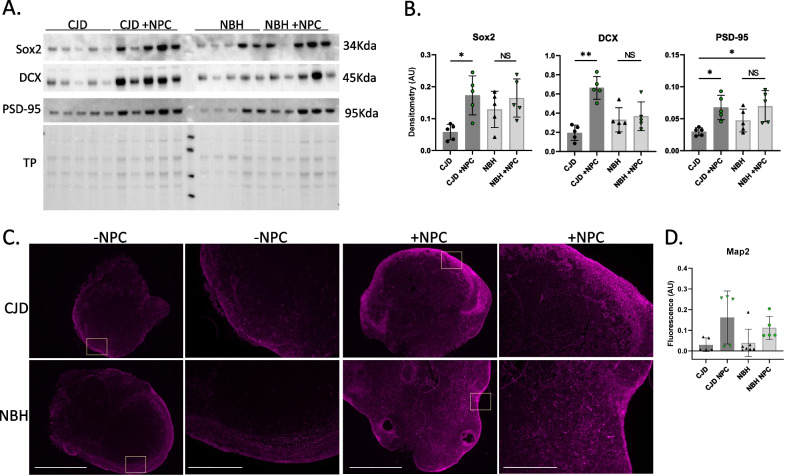
CJD-infected COs seeded with NPCs have higher neuronal linage markers than unseeded CJD-infected organoids. **A** Western blotting for neuronal markers, Sox2, DCX and PSD-95 of CJD-infected unseeded (lanes 1–5) and unseeded (lanes 6–10) and NBH ‘mock’ infected unseeded (lanes 12–16) and seeded (lanes 17–21) organoid lysates after harvest at 182 dpi (un-cropped blot images can be found in Additional file 1: Fig. S4). **B** Quantification of western bolts normalized to total protein (*n* = 5). **C** Immunofluorescence staining for neuronal marker MAP2 with yellow boxes indicating where higher magnification image was taken. Representative scale bars are shown in CJD without NPC and are 1000 µm and 400 µm, respectively. **D** Fluorescent intensity quantification of Map2 staining, with each dot representing an individual organoid (*n* = 5–7). Statistics were preformed using Kruskal–Wallis test with Dunn’s correction for multiple comparisons. *p < 0.05, **p < 0.01, *NS* Not significant

Following collection at the experimental end point, organoids were examined via western blot for detection of Sox2, DCX, and PSD-95 (a post-synaptic marker). This showed that all three markers were significantly increased in the NPC-seeded CJD-infected COs compared with their unseeded counterparts (Fig. 2A & B, uncropped blots are shown in Additional file 1: Fig. S4). Changes in the NPC-seeded NBH controls were variable and not significant. An increased presence of neuronal markers in the NPC-seeded COs was supported by MAP2 immunofluorescence staining (Fig. 2C), which was increased in both the NBH control and CJD-infected organoids (Fig. 2D). Unfortunately, the NPC’s intrinsic GFP did not survive fixation and so we cannot conclude which cells are NPC derived. Regardless, the presence of the seeded NPCs increased the detection of neuronal markers in both the NBH and CJD samples with the sCJD infections showing greater changes.

### Neuroelectrophysiology showed improvements in some parameters in the NPC-seeded sCJD organoids

**Fig. 3:**
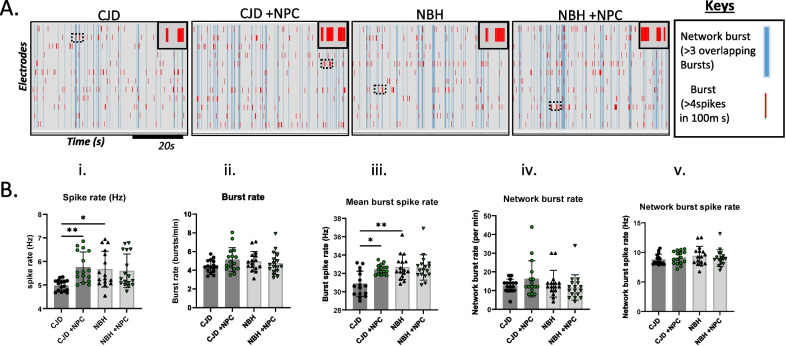
Neuronal network activity in NPC-seeded or unseeded COs following ~ 180 days of inoculation with normal brain homogenate (NBH) or CJD brain homogenate. **A** Raster plots displaying bursts (each red line indicates a burst) and network bursts (each blue line indicates overlapping bursts between > 3 electrodes) over a ~ 60 s recording. The top-right corner inset displays a zoom-in of the bursts within the dotted rectangular box. Keys are displayed on the right panel. **B** Parameters can be calculated from the raster plots that broadly translate to the functioning of neurons in the brain. Neuronal population network activity is measured as (i) spike rate (action potentials), (ii) burst rate (periods of rapid action potential spiking), (iii) spike rate in bursts (average number of spikes per burst), (iv) network burst count (burst occurring in synchrony on multiple electrodes indicating wider neuronal network communication), and v) spike rate in network burst (average action potential spiking in synchronized bursts on multiple electrodes). **B** Average means were compared between groups by the Kruskal–Wallis test with Dunn’s correction for multiple comparisons. Each dot is an “n” representing an organoid (*n* = 15–17). Data are presented as mean ± SD. *p < 0.05, **p < 0.01

To determine if the NPCs could produce any improvement in CO function, neuroelectrophysiology was carried out on whole organoids. At ~ 180 dpi, the overall spike rate was significantly reduced in unseeded CJD-infected COs relative to uninfected controls (Fig. 3Bi.). However, no deficit was observed when measuring the burst rate, network burst rate, and network burst spike rate (Fig. 3A, B ii, iv and v.). In similarity with the overall spike rate, the burst spike rate was significantly reduced by the CJD infection in the unseeded COs (Fig. 3A, Biii). These results suggest a limited degree of neuronal network connectivity damage at this stage of the organoid infections, in which the overall spike rate deficit was likely due to synaptic retraction without neuronal loss. Importantly, NPC seeding significantly rescued the spike rate deficits in CJD-infected COs. The NPC-seeded CJD-infected COs exhibited overall and burst spike rates that were significantly higher than the unseeded CJD COs and no longer different from the NBH controls (Fig. 3A, B i and iii). We additionally measured these parameters at the mid-incubation period (105–122 dpi and 15–32 days post-seeding); however, no significant differences were observed in neuroelectrophysiological function at this earlier time (Additional file 1: Fig. S5A & B). Therefore, following 180 days of CJD infection in COs, neuronal firing became dysfunctional, which was rescued by NPC treatment.

### Influence of NPCs on prion disease parameters

**Fig. 4:**
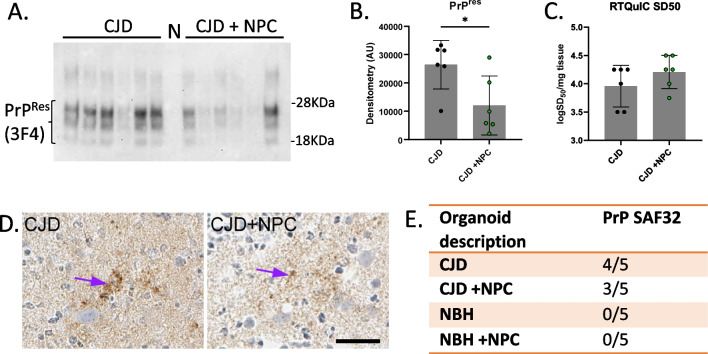
Effect of NPC seeding on CO infection parameters. **A** Western blot of proteinase K (PK) digested PrP from the CJD infections using 3F4 anti-PrP antibody (uncropped blots are shown in Additional file 1: Fig. S6). Samples were PK treated with 10 µg/ml. Lane N is NBH + NPC. **B** Quantification of PrP^Res^ western blot intensities. **C** RT-QuIC 50% seeding dose (SD50) for CJD samples with or without NPC (*n* = 6). **D** Example PrP histochemistry of the NPC unseeded and seeded organoids with SAF32 antibody. Purple arrows show deposits. Scale bar is 25 µm **E** IHC summary of abnormal PrP deposits. With number scoring positive over total number of organoids per group examined

Western blotting for protease-resistant PrP (PrP^Res^), which is an indicator of propagation and accumulation of infectious prions, showed increased detection in the unseeded CJD-infected compared with the NPC-seeded CJD-infected COs (Fig. 4A & B, uncropped blots are shown in Additional file 1: Fig. S6). Real-time Quaking Induced Conversion (RT-QuIC) is a sensitive assay for measuring the presence of prions by using their propensity to template conversion of more mis-folded PrP. Despite detecting less PrP^Res^ in the NPC containing sCJD-infected COs, no significant differences were observed by RT-QuIC (Fig. 4C). Immunohistochemistry staining for PrP showed a small amount of deposition in most of the organoids but no significant difference between organoids seeded with NPCs and those left unseeded (Fig. 4D and E). Organoids that received NBH had no PrP^Res^, no seeding activity and no abnormal deposits by IHC. Glial fibrillary acidic protein (GFAP), indicative of astrocytes, and superoxide dismutase 1 (SOD1), indicative of oxidative stress, showed a small decrease by western blot in the NPC-seeded CJD-infected organoids compared with the unseeded CJD-infected COs (Additional file 1: Fig. S7A & B). Overall, NPC seeding of the sCJD-infected organoids produced modest improvements in the biochemical parameters of infection.

### NPCs increase the detection of cell signaling proteins

**Fig. 5:**
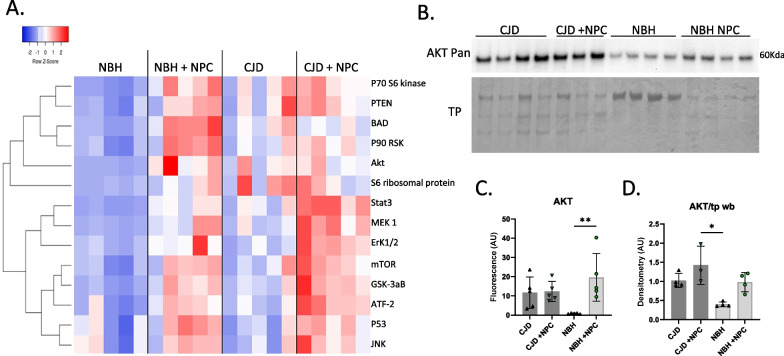
Analysis of cell signaling intermediates changed as a result of infection and NPC seeding. **A** Heat map of multiplex immunoassay data for signal transduction intermediates showing Z-scores of 5 organoids per treatment. **B** Western blotting of pan AKT for validation of the Bioplex signaling array data (un-cropped blot images can be found in Additional file 1: Fig. S8). **C** Bioplex AKT quantification (mean fluorescence normalized to total protein). **D** Quantification of western blot AKT (Pan) normalized to total protein (Coomassie). Markers on graphs represent individual organoids with shaded bars showing the mean and error bars showing the SD. Statistics were preformed using Kruskal–Wallis test with Dunn’s correction for multiple comparisons. **p < 0.01, *p < 0.05

To further investigate potential improvement in cellular functions, we investigated cell signaling intermediates using MAPK 9-plex and AKT 8-plex signal transduction panels. Although a few increases in cell signaling pathways were seen in the CJD-infected COs, these appeared to be variable across organoids and not significant (Fig. 5A). The largest changes appeared to have been induced by the addition of the NPCs regardless of infection status (Fig. 5A) and might be linked with NPC integration and maturation. Bioplex data were validated with western blot for pan AKT, which showed similar trends (Fig. 5B, C, & D, uncropped blots are shown in Additional file 1: Fig. S8). Thus, it appears the addition of the NPCs significantly increased expression of proteins associated with cellular signal transduction pathways unrelated to CJD infection.

## Discussion

Herein, we sought to investigate the potential of NPCs to rescue prion infection parameters, especially functional readouts, in a completely human, cerebral organoid model of structured brain tissue. Our findings show that while the beneficial effects of NPC addition were mild, they were significant, increasing the detection of neuronal markers in the organoids as well as improving certain neuroelectrophysiological parameters. One of the long-standing problems in the field of prion diseases is finding a treatment approach that might show therapeutic efficacy. Compounds that stop prion propagation may be effective if administered early enough to prevent significant damage but after damage has occurred a different approach is needed to attempt to restore brain function and therefore patient quality of life. Our results indicate that NPCs have the potential to restore some function in COs that have established prion disease.

Aside from the highly invasive procedure used to introduce the cells, when a similar approach was trialed in a Parkinson’s disease patient other limiting factors were identified, such as costs and survival of cell grafts. Examination of test grafts in mice found that allogenic grafts were rejected within 2 weeks, whereas autologous cells survived [13]. While we found an increase in LDH with NPC treatment, COs did retain NPC cells throughout experimentation. However, it is possible that we would have seen a more dramatic improvement in function and disease parameters if we had varied the seeded cell numbers or followed up with more than one treatment. Although costly and time intensive, especially if cells are produced from each individual patient, the ability to manipulate treatment cells can offer an advantage. For instance, the possibility of using PrP knock-out cells has previously been investigated by Relaño-Ginés and colleagues [17]. Although the knock-out cell therapy appeared to offer little benefit over using wild type cells [17], more general knock-down of PrP has previously demonstrated beneficial effects on prion disease survival and pathogenesis in mice [30–32]. As the single critical factor for progression of prion diseases is expression of PrP, NPCs engineered to have low or no PrP expression may be useful for preventing further prion propagation, and with CRISPR technology now widely available is a reasonable consideration. Another cell engineering option was developed by Fujita et al., where the authors expressed an anti-PrP antibody fragment in an engraftable murine microglial cell line and found that prophylactic or early engraftment of the cells could extend murine scrapie survival times [33]. Potentially, NPCs could be engineered to produce such fragments providing a ‘double-hit’ against disease progression and dysfunction. Antibody therapy has been trialed in human patients and was found to be well tolerated reaching the desired CSF concentration in four out of six patients [34]. While these studies are ongoing to determine the therapeutic benefit, they support that antibody therapy, in the context of prion disease, would likely be non-toxic and, therefore, alternative delivery mechanisms could be worth investigating.

While the approach we took was to generate NPCs from iPSCs, which would allow autologous matching of the NPCs to the patient, other approaches might also be possible. It may be feasible to engineer commercially available neuroprogenitor cells to be indistinguishable to self, permitting many transplants to be possible from a less expensive source than re-programming cells from each individual patient. Mesenchymal stem cells also offer an option and IC or IV administration of these has improved murine survival in prion disease [15, 16], and intranasally delivered mesenchymal stromal cells decreased neuroinflammation and astrogliosis, although no increase in survival was observed [18]. Mesenchymal cells have additionally been used to treat amyotrophic lateral sclerosis patients by intrathecal injection. They were safe and tolerated by the patients, with several patients showing clinical benefits [35, 36]. Thus, these may also offer an alternative approach that could be beneficial and can be tested in the human CO model.

Another similar approach where NPC grafting may contribute to therapeutic benefit could be combination therapy. This has been investigated in a mouse model of genetic CJD caused by the E200K mutation. The health and lifespan of these mice were significantly extended using a combination of NPC engraftment and a nanodroplet formulation of pomegranate seed oil with antioxidant properties [19, 20]. Strategies to target the mis-folding process directly in conjunction with improving neuronal network function have the potential to both halt or slow prion propagation while simultaneously repairing some of the disease damage.

The CO model offers a power approach for investigating the pathogenesis of human brain disease and the potential for success of putative treatments. However, both the approach and the organoid model are not without limitations. Herein, we tested NPC treatment on only one sCJD infection of organoids from one single donor. Future directions will consider different sCJD infections to account for different disease subtypes and different donor iPSCs to ensure the recovery of function was not donor specific. Limitations of the organoid model includes the fetal nature of the differentiated tissues that, while supportive of the propagation of infectious prions, may not be able to fully recapitulate the features of a disease that affects older individuals. The limitations of the system have been discussed in more extensive detail elsewhere [37].

## Conclusions

Improving patient quality of life is a greatly sought-after aim when developing therapeutics for neurological diseases; extending life with no functional recovery offers little benefit for patients or their care givers. Herein, we show that NPC supplementation into COs with established prion infection can improve neuronal network function. While more research is needed to optimize delivery of cell therapies without invasive transplantation, the demonstration of some functional recovery in a human CJD model should provide optimism that an approach directed to improve patient quality of life can succeed where traditional anti-prion compounds have been limited.

### Supplementary Information

**Additional file 1: Fig. S1.** PPS neuroelectrophysiology.** Fig. S2.** Organoid characterisation.** Fig. S3.** Un-cropped western blots.** Fig. S4.** Un-cropped western blots.** Fig. S5.** Additional organoid electrophysiology.** Fig. S6.** Uncropped western blots.** Fig. S7.** GFAP and SOD1 western blotting.** Fig. S8.** Un-cropped western blots.

## Data Availability

The datasets used and/or analyzed during the current study are available from the corresponding author on reasonable request.

## Abbreviations

CO: Cerebral organoid
Dpi: Days post-infection
iPSC: Induced pluripotent stem cells
NPC: Neural progenitor cell
PrP: Prion protein
sCJD: Sporadic Creutzfeldt-Jakob disease
