# Supplementary material for: Neural cell engraftment therapy for sporadic Creutzfeldt-Jakob disease restores neuroelectrophysiological parameters in a cerebral organoid model
Source: Stem Cell Res Ther. 2023 Dec 5;14:348. doi: 10.1186/s13287-023-03591-2 (PMC10696693; doi:10.1186/s13287-023-03591-2)

***Supplementary Figure 1.*** *PPS treatment does not improve neuroelectrophysiological dysfunction in sCJD infected organoids.* A. Schematic depicting the PPS treatment and multi-electrode array (MEA) reading times. B. Quantification of the spike rates with and without 3 µg/ml PPS treatment after 48 hrs and 6 weeks. The average spike rate was compared between the PPS-treated and untreated groups by an unpaired nonparametric Student's t-test (Mann-Whitney test).


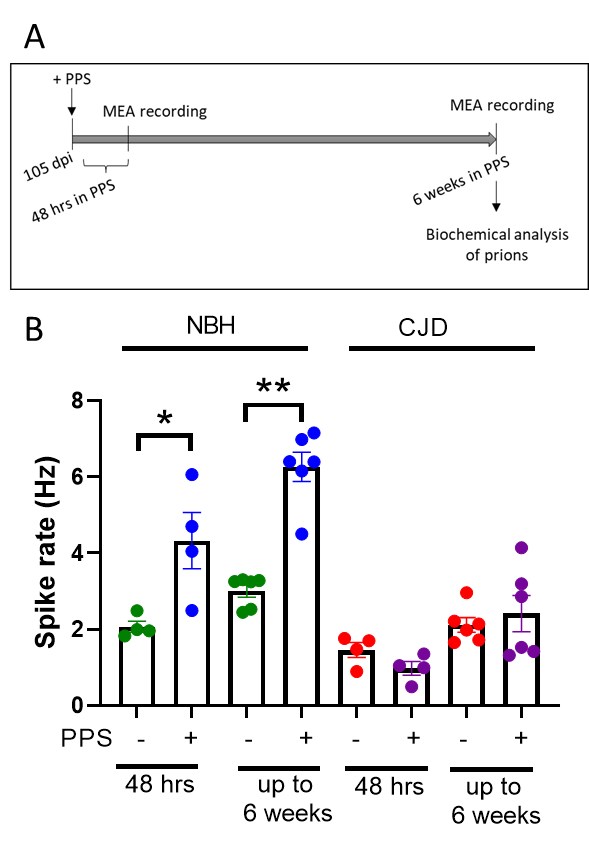


***Supplementary Figure 2.*** *General cerebral organoid characterization of 6- to 11-month-old organoids.* IHC shows Sox2 (immature progenitor marker) at 7 months with immature neuron (DCX), mature neuron (NFL & Map2), and astrocyte (GFAP) markers at 11 months old. Immunofluorescence shows higher magnification of mature neuronal markers (NFL & β-tubulin) at 6 months old.


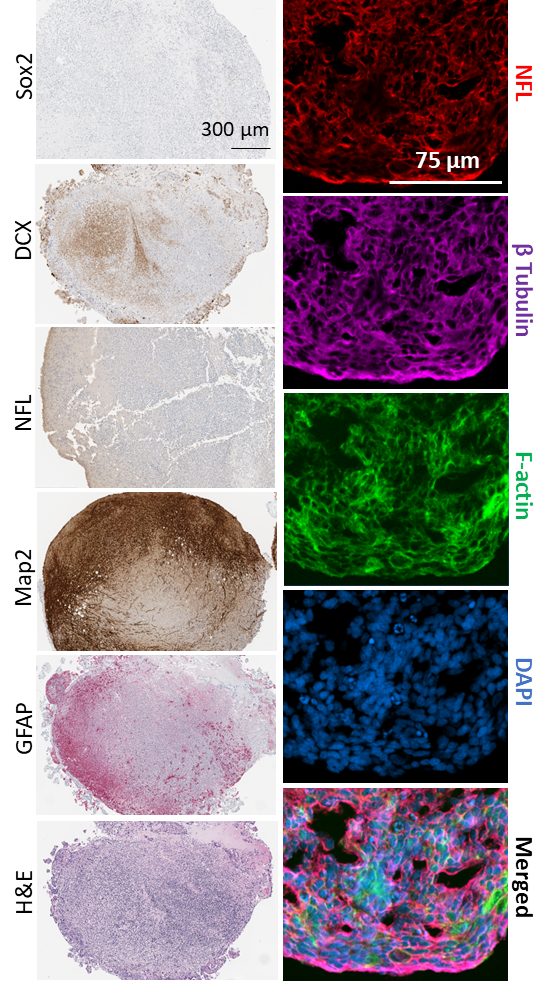


***Supplementary Figure 3.*** *Un-cropped western blots.* Blots shown in Figure 1B.


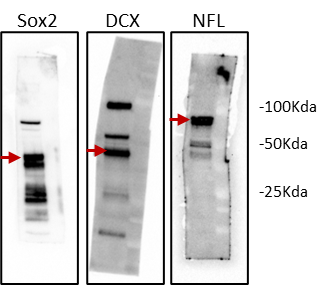


***Supplementary Figure 4.*** *Un-cropped western blots.* Blots shown in Figure 2. X = lanes not used in this experiment.


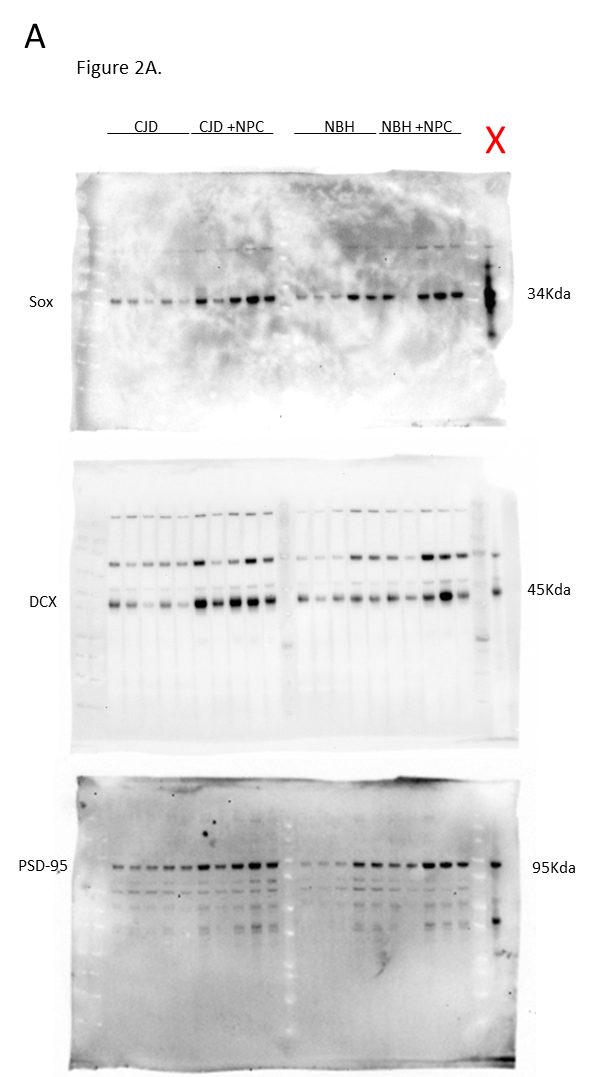


***Supplementary Figure 5.*** *No neuronal network activity differences are seen between COs without and with NPC (~20 days post treatment) and following ~110 days of inoculation with normal brain homogenate (NBH) or CJD brain homogenate.* (A) Raster blots displaying bursts (each red line indicates a burst) and network bursts (each blue line indicates overlapping bursts between >3 electrodes) over a ~60-second recording. The top-right corner inset displays a zoom-in of the bursts within the dotted rectangular box. Keys are displayed on the right panel. (B) Neuronal network activity is measured as spike rate (i.), burst rate (ii.), spike rate in bursts (iii.), network burst count (iv.), and spike rate in network burst (v.).


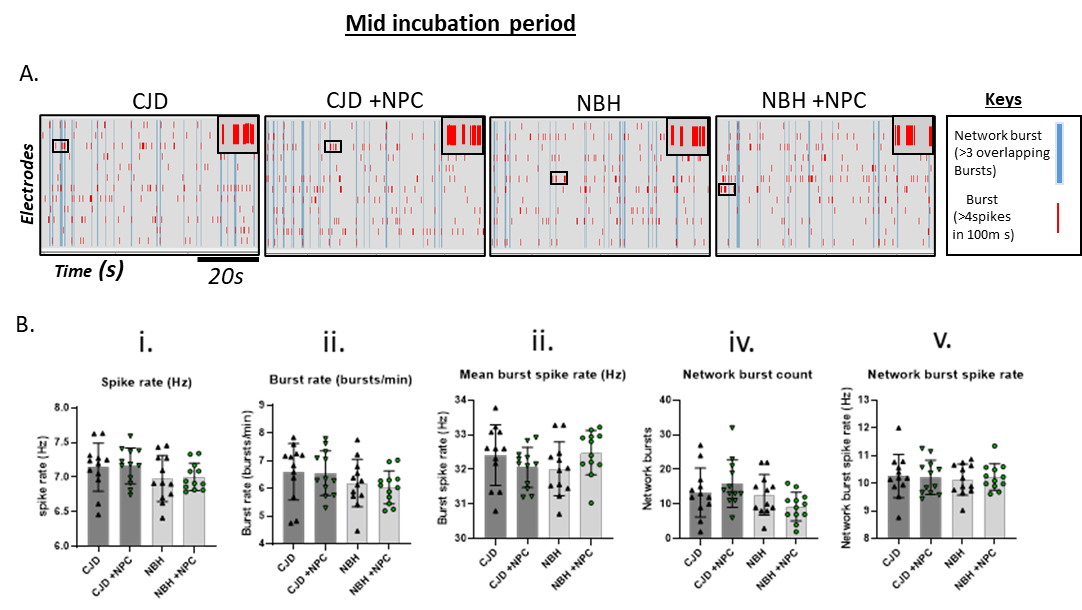


***Supplementary Figure 6.*** *Un-cropped western blots.* Blots shown in Figure 4A.

***
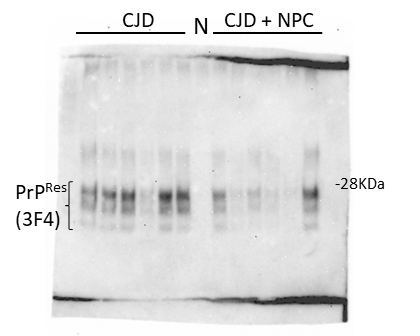
***

***Supplementary Figure 7.*** *A. Western blots for GFAP and SOD1 and B. corresponding quantification.* All signals are normalized to total protein (Coommassie). Single points on graphs represent an individual organoid with shaded bars showing the mean and error bars showing the SD.


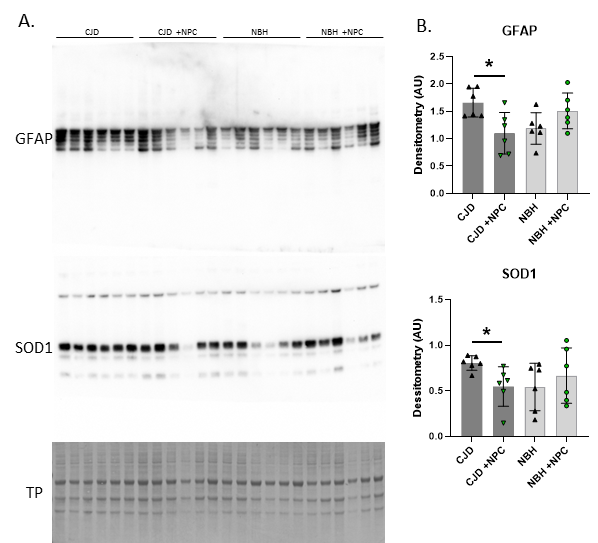


***Supplementary Figure 8.*** *Un-cropped western blots.* Blots shown in Figure 5. X = lanes not used in this experiment.


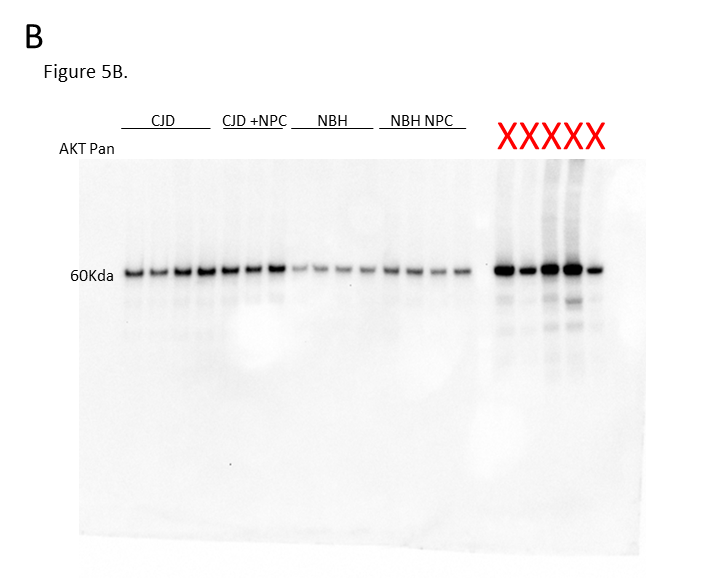

Supplement: Supplementary file 1 — Additional file 1: Fig. S1. PPS neuroelectrophysiology. Fig. S2. Organoid characterisation. Fig. S3. Un-cropped western blots. Fig. S4. Un-cropped western blots. Fig. S5. Additional organoid electrophysiology. Fig. S6. Uncropped western blots. Fig. S7. GFAP and SOD1 western blotting. Fig. S8. Un-cropped western blots. [file 13287_2023_3591_MOESM1_ESM.docx]
